# Outbreak of Shiga toxin-producing *Escherichia coli* (STEC) O26 paediatric haemolytic uraemic syndrome (HUS) cases associated with the consumption of soft raw cow’s milk cheeses, France, March to May 2019

**DOI:** 10.2807/1560-7917.ES.2019.24.22.1900305

**Published:** 2019-05-30

**Authors:** Gabrielle Jones, Sophie Lefèvre, Marie-Pierre Donguy, Athinna Nisavanh, Garance Terpant, Erica Fougère, Emmanuelle Vaissière, Anne Guinard, Alexandra Mailles, Henriette de Valk, Marc Fila, Corentin Tanné, Caroline Le Borgne, François-Xavier Weill, Stéphane Bonacorsi, Nathalie Jourdan-Da Silva, Patricia Mariani-Kurkdjian

**Affiliations:** 1 Santé publique France, Saint-Maurice, France; 2 Institut Pasteur, Centre National de Référence des *Escherichia coli, Shigella* et *Salmonella*, Paris, France; 3 French Ministry of Agriculture, Agrifood and Forestry, Paris, France; 4 Santé publique France, Auvergne-Rhône-Alpes region, France; 5 Santé publique France, Occitanie region, France; 6 Paediatric Nephrology Unit, CHU Arnaud de Villeneuve-Université de Montpellier, Montpellier, France; 7 Centre de Référence des Maladies Rénales Rares, Hôpital Femme Mère Enfant, Bron, France; 8 Faculté de Médecine Lyon Est, Université Lyon 1, Lyon, France; 9 French Ministry of Health, Paris, France; 10 Department of Microbiology, Robert-Debré Hospital, AP-HP, Paris, France

**Keywords:** Escherichia coli, STEC, HUS, O26, raw milk cheese, France

## Abstract

We report an outbreak of Shiga toxin-producing *Escherichia coli* (STEC) associated paediatric haemolytic uraemic syndrome linked to the consumption of raw cow’s milk soft cheeses. From 25 March to 27 May 2019, 16 outbreak cases infected with STEC O26 (median age: 22 months) were identified. Interviews and trace-back investigations using loyalty cards identified the consumption of raw milk cheeses from a single producer. Trace-forward investigations revealed that these cheeses were internationally distributed.

## Outbreak detection

From 25 March to 27 April 2019, 19 suspected Shiga toxin-producing *Escherichia coli* (STEC) associated paediatric haemolytic uraemic syndrome (HUS) cases were notified by French hospital paediatric departments to Santé publique France, compared with 5–10 cases during the same period in previous years [1]. Thirteen cases were confirmed as serogroup O26, with whole genome sequencing (WGS) underway for strain comparison. Initial epidemiological investigations using a trawling questionnaire identified the consumption of raw cow’s milk soft cheeses (Saint-Félicien and Saint-Marcellin) as the common link for eight of these 13 cases. Trace-back investigations using supermarket loyalty cards identified a common producer (producer A) of these cheeses for three cases and on the basis of this information a recall was initiated by French health authorities on 27 April 2019 [2].

## Outbreak investigations

Box **Case definition for STEC associated paediatric HUS cases, France, March–May 2019**

• A suspected case was defined as a child aged less than 15 years with HUS and symptom onset after 1 March 2019

• An outbreak case was defined as infection with serogroup O26 STEC, belonging to the outbreak cluster defined by the French National Reference Centre for *Escherichia coli*, *Shigella* and *Salmonella* using whole genome sequencing

HUS: haemolytic uraemic syndrome; STEC: Shiga toxin-producing *Escherichia coli.*

**Figure 1:**
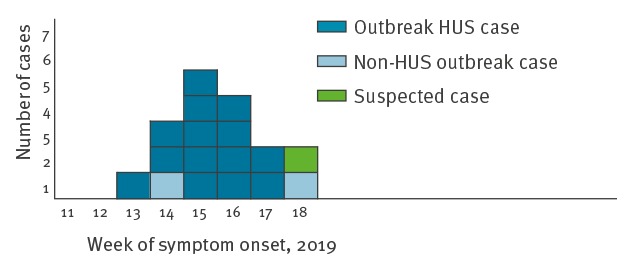
Distribution of outbreak and suspected cases by week of symptom onset, France, March–May 2019 (n = 17)

As at 27 May 2019, investigations identified 16 outbreak cases including 14 paediatric HUS cases and two cases with uncomplicated diarrhoea (one child and one adult). Investigations are ongoing for one suspected case. The 16 outbreak cases reside in six administrative regions in France. All paediatric cases are under 5 years of age; the median age is 22 months (overall age range: 6 months–63 years). Eight cases are female. Date of symptom onset was between 31 March (week 13) and 29 April (week 18) (Figure 1). All HUS cases were hospitalised. Thirteen cases received blood and/or platelet transfusion and seven underwent haemodialysis. Six cases had neurological complications, all of them received transfusions and three also had haemodialysis.

Hus: haemolytic uraemic syndrome.

The families of all 16 outbreak cases and the suspected case were interviewed about their at-risk exposures during the 10 days before symptom onset. Families of 16 cases (15 outbreak cases and one suspected case) reported the consumption of Saint-Félicien or Saint-Marcellin raw cow’s milk cheeses by either the case (n = 12) or household members (n = 4). One outbreak case did not report consumption of these cheeses. For the 16 cases with reported consumption of these cheeses, trace-back investigations using loyalty cards and supply data from the different shops where the caretakers reported purchasing the cheeses identified a link with producer A for 13 (all outbreak cases).

Producer A manufactured only Saint-Félicien and Saint-Marcellin cheeses. To date, no positive STEC O26 cheese or milk samples have been identified. Investigations, including sampling of the cheeses and trace-back of the milk supply chains, are ongoing. 

Four outbreak cases had not consumed the cheeses themselves but a household member had. This suggests the affected child may have been infected via cross contamination (knives, cutting board, hands, etc.). None of the household members reported symptoms of illness, indicating that the cases were unlikely to have been infected by person-to-person transmission. Investigations are ongoing in an attempt to further document the exposures of these cases (consumption of cheeses or other food items cut by the knives or on the same cutting board as the suspected cheeses). Only one in 16 outbreak cases reported a family member with self-limiting diarrhoea (no stool analysis).

## Microbiological investigations

In France, STEC surveillance is based on surveillance of HUS in children aged less than 15 years by a network of voluntary paediatric and paediatric nephrology departments [1]. All medical laboratories in France can send stool samples to the associated laboratory of the National Reference Centre (NRC-RD) for *E. coli* (Microbiology department, Robert Debré Hospital, Paris) for STEC screening, confirmation and determination of serogroup and virulence factors.

Since April 2017, isolated strains are sent to the French National Reference Centre for *Escherichia coli*, *Shigella* and *Salmonella* (NRC-ESS) at Institut Pasteur (Paris, France) for whole genome sequencing (WGS) and determination of cluster affiliation. WGS is performed as previously described [3]. Stool samples for all suspected cases were sent to the NRC-RD and isolated strains underwent WGS at the NRC-ESS.

The determination of serotype (O and H antigens) [4], virulence genes (*stx, eae, ehxA, saa, aggR and subA* genes), acquired resistance genes and MLST are performed using tools available at the Center for Genomic Epidemiology (https://cge.cbs.dtu.dk/services/). Phylogenetic analysis is performed by single nucleotide polymorphism (SNP) and core genome multilocus sequence typing (cgMLST) analysis integrated into Enterobase [5].

**Figure 2:**
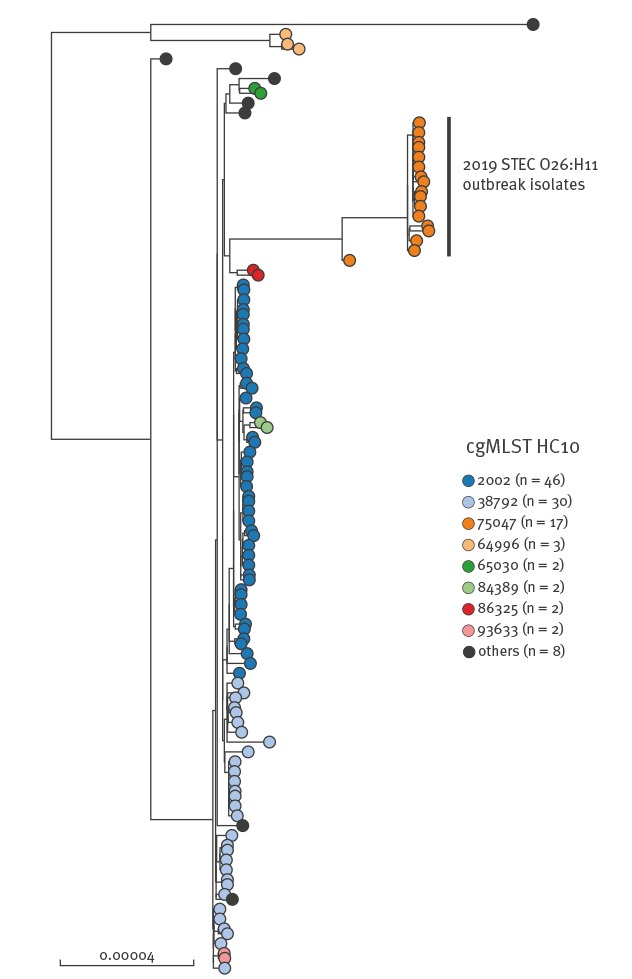
SNP-based phylogenetic tree of STEC O26:H11, ST21, performed by the NRC-ESS, France, 2019

SNP analysis revealed that 16 human isolates of *E. coli* serotype O26:H11 harboring *stx2a* and *eaeβ* virulence genes, but not the *ehxA* gene, clustered tightly together (Figure 2). Clustered isolates displayed MLST type ST21 and an identical type ‘75047’ by hierarchical clustering of cgMLST data differing by < 10 alleles (HC10).

cgMLST: core genome multilocus sequence typing; NRC-ESS: French National Reference Centre for *Escherichia coli*, *Shigella* and *Salmonella*; STEC: Shiga-toxin producing *Escherichia coli*; SNP: single nucleotide polymorphism.

The phylogenetic analysis was carried out by using the « create SNP project » tool in Enterobase on 105 French STEC genomes belonging to cgMLST type HC20|2002, with the isolate 201902833 as reference. The tree was based on 2,463 SNPs found in non-repetitive regions that are present in 97% or more of all the queried genomes. The tips are coloured according to the cgMLST type HC10. The scale bar refers to the frequency of mutation per site in the core genome (this corresponds to approximately 180 SNPs).

Of all the human isolates received at the NRC-ESS from 2016 to April 2019, only one (201808628) belonged to the same cgMLST HC10|75047 cluster. SNP analysis revealed that this isolate was a close ancestor to the current 2019 outbreak isolates. The strain was isolated in September 2018, from case of paediatric HUS residing in the neighbouring administrative department from producer A. Investigations did not identify a possible link with current outbreak cases. All studied genomes were deposited into EnteroBase and raw reads of three representative outbreak isolates (201902616, 201902803 and 201902835) were also deposited to the European Nt Archive (https://www.ebi.ac.uk/ena), under study accession number PRJEB32463.

## Control measures

Based on the initial results of epidemiological and trace-back investigations (cgMLST results and microbiological analysis of the cheeses were pending), the complete production of the incriminated producer was recalled on 27 April [2]. The reasons for recalling at a very early stage were: (i) the high proportion of interviewed cases reporting consumption of these cheeses (8/13 STEC O26 cases initially under investigation), (ii) the rapid identification of a common producer of the cheeses consumed by three cases and, (iii) the severity of the infections in the cases. All cheeses from the producer A made from 1 February to 27 April 2019 were recalled from supermarkets and retail shops. Investigations are ongoing at producer A facilities and microbiological analyses of milk and cheese samples are ongoing.

On a national level, a press release regarding the recall was issued on 27 April [2] to inform consumers about the list of brands under which these cheeses were sold. People who still had cheese from this producer in their home were advised to not consume it and return it to the place of purchase. On 2 May, a second press release was issued with an updated list of potential brands under which the suspected products were sold [6]. Recommendations about the risk of consumption of raw milk cheeses in children, especially those aged less than 5 years, were diffused in the media (press release, newspapers).

Information was relayed to international health authorities through a message on the European Centre for Disease Prevention and Control (ECDC) Epidemic Intelligence Information System for Food- and Waterborne Diseases and Zoonoses (EPIS-FWD) published on 30 April. Representative sequences were made available on 7 May. As at 27 May, none of the 13 responding countries have reported cases linked to this outbreak.

Trace-forward investigations identified the export of the recalled cheeses to countries inside and outside the European Union. The information was shared via a Rapid Alert System for Food and Feed (RASFF: notification 2019.1615) message and an International Food Safety Authorities Network (Infosan) message on 30 April [7]. As at May 27, 33 countries had received distributions of the incriminated products.

## Conclusion

This outbreak of paediatric HUS in France was linked to the consumption of Saint-Marcellin and Saint-Félicien cheeses. While paediatric HUS cases are likely to be notified to public health authorities through the national surveillance system, STEC infections presenting as non-complicated diarrhoea may not be identified (no stool analysis, samples not sent to the NRC-RD) and the number of cases linked to this outbreak may be underestimated. The outbreak is notable for the young age of cases and the severity of the illness. These factors contributed to the decision to recall the products early in the investigation, despite pending results of the cgMLST and case/product investigations. Timely notification of cases by the French paediatric HUS surveillance system enabled rapid epidemiological investigations during which crucial information to guide control measures was obtained. Investigations are ongoing and it is possible that additional cases may be notified, as information about the product recall may not have reached some consumers in France or in countries where the cheeses were imported.

In the last 10 years, three HUS outbreaks linked to the consumption of raw milk cheeses have occurred in France, including two in 2018 that were linked to the consumption of Reblochon cheese [8-10]. In the current outbreak, several families reported consumption of the suspected cheeses by family members, but not by the ill child. This suggests that the risk of cross contamination from food vehicles consumed by family members of young children should also be considered during investigations.

These outbreaks highlight the risk of consuming raw milk cheese, particularly for young children. Increasing public awareness of the risk is therefore an important preventive measure. An interdisciplinary group of public health and food safety authorities is currently working on development of communication strategies in France to improve consumer awareness regarding these risks.
